# Dental Infection of *Porphyromonas gingivalis* Induces Preterm Birth in Mice

**DOI:** 10.1371/journal.pone.0137249

**Published:** 2015-08-31

**Authors:** Min Ao, Mutsumi Miyauchi, Hisako Furusho, Toshihiro Inubushi, Masae Kitagawa, Atsuhiro Nagasaki, Shinichi Sakamoto, Katsuyuki Kozai, Takashi Takata

**Affiliations:** 1 Department of Oral and Maxillofacial Pathobiology, Institute of Biomedical and Health Sciences, Hiroshima University, Hiroshima, 734–8553, Japan; 2 Department of Pediatric Dentistry, Institute of Biomedical and Health Sciences, Hiroshima University, Hiroshima, 734–8553, Japan; 3 Center of Oral Clinical Examination, Hiroshima University Hospital, Hiroshima University, Hiroshima, 734–8553, Japan; Xavier Bichat Medical School, INSERM-CNRS - Université Paris Diderot, FRANCE

## Abstract

**Background:**

Epidemiological studies have revealed a link between dental infection and preterm birth or low birth weight (PTB/LBW), however, the underlying mechanisms remain unclear. Progress in understanding the associated mechanisms has been limited in part by lack of an animal model for chronic infection-induced PTB/LBW, mimicking pregnancy under conditions of periodontitis. We aimed to establish a mouse model of chronic periodontitis in order to investigate the link between periodontitis and PTB/LBW.

**Methods:**

To establish chronic inflammation beginning with dental infection, we surgically opened mouse (female, 8 weeks old) 1st molar pulp chambers and directly infected with w83 strain *Porphyromonas gingivalis* (*P*.*g*.), a keystone periodontal pathogen. Mating was initiated at 6 wks post-infection, by which time dental granuloma tissue had developed and live *P*.*g*. was cultured from extracted tooth root, which serves as a persistent source of *P*.*g*. The gestational day (gd) and birth weight were recorded during for *P*.*g*.-infected and control mice, and serum and placental tissues were collected at gd 15 to evaluate the systemic and local conditions during pregnancy.

**Results:**

Dental infection with *P*.*g*. significantly increased circulating TNF-α (2.5-fold), IL-17 (2-fold), IL-6 (2-fold) and IL-1β (2-fold). The *P*.*g*.-infected group delivered at gd 18.25 vs. gd 20.45 in the non-infected control (NC) group (p < 0.01), and pups exhibited LBW compared to controls (p < 0.01). *P*.*g*. was localized to placental tissues by immunohistochemistry and PCR, and defects in placental tissues of *P*.*g*. infected mice included premature rupture of membrane, placental detachment, degenerative changes in trophoblasts and endothelial cells, including necrotic areas. *P*.*g*. infection caused significantly increased numbers of polymorphonuclear leukocytes (PMNLs) and macrophages in placental tissues, associated with increased local expression of pro-inflammatory mediators including TNF-α and COX-2. Further placental tissue damage was indicated in *P*.*g*. infected mice by decreased CD-31 in endothelial cells, increased expression of 8OHdG, an indicator of oxidative DNA damage, and cleaved caspase-3, a marker of apoptosis. *In vitro*, *P*.*g*. lipopolysaccharide significantly increased expression of COX-2, IL-8 and TNF-α, in HTR-8 trophoblasts in an NF-κB-dependent fashion.

**Conclusions:**

Our novel mouse model supports previous epidemiological studies signifying dental infection as predisposing factor for PTB/LBW. We demonstrate PTB and LBW in infected mice, translocation of *P*.*g* to placental tissues, increased circulating and local pro-inflammatory markers, and the capability of *P*.*g*. LPS to directly induce cytokine production in trophoblasts, *in vitro*. These findings further underscore the importance of local and systemic infections and inflammation during pregnancy and suggest that prevention and/or elimination of dental infections such as marginal or periapical periodontitis before pregnancy may have a beneficial effect on PTB/LBW.

## Introduction

Preterm birth (PTB) and low birth weight (LBW) are the most common causes of morbidity and mortality in newborn infants [1]. In 2010, an estimated 11.1% of all live births worldwide were preterm, ranging from about 5% of births in Japan and several European countries, up to 18% of births in some African countries [2]. PTB is associated with 75% of perinatal mortality and more than half of the long-term morbidity, including conditions such as cerebral palsy, bronchopulmonary dysplasia, intraventricular hemorrhage, necrotizing enterocolitis, seizures and sepsis [3]. The World Health Organization (WHO) aims to reduce the frequency of PTB/LBW, and increasing evidence suggests an association between mild chronic inflammation and PTB [4]. It has been demonstrated that intrauterine infection increases the production of prostaglandins, tissue necrosis factor (TNF)-α, interleukin (IL)-6 and IL-8, leading to more frequent and intense uterine contractions, in turn contributing to preterm labor [5,6,7].

Chronic periodontitis, the most common form of periodontitis, is primarily caused by Gram-negative periodontal pathogens and the host inflammatory response [8]. Periodontal pathogens are able to enter the bloodstream and become disseminated through whole body [9]. In 1996, results of a case-control study suggested that maternal periodontal disease caries a 7-fold increased factor for PTB/LBW [10]. Furthermore, in pregnant women at high-risk of premature labor, periodontal pathogens were detected in both the periodontal pocket and the amniotic fluid [11], and antigens of *Porphyromonas gingivalis* (*P*.*g*.), a keystone periodontal pathogen have been detected in placental tissues of women with chorioamnionitis [12].

Mechanisms linking periodontitis to PTB/LBW and adverse pregnancy outcomes remain unclear, in part due to lack of an animal model for chronic infection-induced PTB/LBW, mimicking pregnancy under conditions of periodontitis. We aimed to establish an experimental mouse model for chronic dental infection-induced PTB/LBW and investigate the link between them.

## Materials and Methods

### Animals

This study was carried out in strict accordance with the recommendations in the Guide for the Care and Use of Laboratory Animals of the Hiroshima University Animal Research Committee and AVMA Guidelines on Euthanasia. The protocol described below was approved by the Committee on the Ethics of Animal Experiments of the Hiroshima University (Permit Number: A09-89). All mice were housed in a specific pathogen free facility in 12 hr light-dark cycles with access to water and food *ad libitum* and health monitoring was conducted every day. Cervical dislocation euthanasia of all adult mice and pups was performed after isoflurane sedation.

### Dental infection by *Porphyromonas gingivalis*

A group of 8 wks old female C57Bl/6J mice (Charles River Japan, Inc., Yokohama, Japan), (n = 52) were randomly divided into two groups that received *P*.*g*. infection or did not, the *P*.*g*.-infected group or negative control (NC) groups (n = 26 each), respectively. Among them, 20 mice were randomly chosen to record gestational day and the remaining 6 were used to collect serum and placental tissue. Dental infection was induced using of the W83 strain of *P*.*g*. as described previously [13,14]. *P*.*g*. was cultured on a sheep blood agar plate using the Anaeropack system (Mitsubishi GasChemical, Tokyo, Japan) at 37°C. After a 2-day incubation, *P*.*g*. was inoculated in 40 ml of trypticase soy broth supplemented with 1% yeast extract, hemin (200 μg), and menadione (20 μg) using the Anaeropack system at 37°C. *P*.*g*. was harvested in the exponential growth phase and CFU (colony-forming unit) was determined at an optical density of 660nm. All the surgeries were performed under intraperitoneal anesthesia with (Pentbarbital sodium: 1.62mg/30g, Kyoritsu Seiyaku Co., Tokyo, Japan) and atropine sulfate (12.5ug/30g, Mitsubishi Tanabe Pharma Co., Osaka, Japan) in the biosafety cabinet of Hiroshima University Animal Facility and maximum efforts were made to minimize the pain and suffering. Briefly, the pulp chambers of the maxillary first molars on the left and right sides were opened with #1/2 round burr. After removing the coronal pulp, a small cotton swab containing 10^8^ CFU of *P*.*g*. W83 strain was inserted into the pulp chamber and sealed with a Caviton (GC Co., Tokyo, Japan).

Mating was initiated at 6 wks post-infection (14 wks-old). Mating males with females was done for up to one week and appearance of vaginal plugs was used to determine gestational day (gd) 0 according to the conventional method [15]. Females that did not show pregnancy within one week were excluded from experiments. Gestational day and birth weight were measured for *P*.*g*.-infected and control groups. Additionally, placental tissues were harvested from gd 15 mice and stored at -80°C for RNA isolation or fixed in 10% neutral buffered formalin for histology. Serum from mice at 0 and 6 wks after infection were also collected and stored at -80°C.

### Histology

Placental tissues were fixed in 10% neutral buffered formalin for 24 hours. Samples were processed and embedded for paraffin sectioning to obtain sections of 4.5 μm in thickness. Standard H&E (hematoxylin and eosin) staining was performed for placenta structural observation. Immunohistochemistry was performed as described previously [16]. Staining was visualized using DAB Peroxidase (HRP) Substrate Kit (DAKO Japan, TOKYO, JAPAN) to produce a brown reaction product indicating antigen localization. Localization of *P*.*g*. in the placental tissue was analyzed by imunohistochemistry using rabbit antiserum against whole *P*.*g*. (1:1,000 dilution, kindly provided by Prof. Kazuyuki Ishihara, Tokyo Dental College, Japan). Anti-mouse CD-31 monoclonal antibody (1:100 dilution, dianova GmbH, Hamburg, Germany) was used for immunohistochemical detection of endothelial cells in placental tissue. Primary antibodies were used to localize the marker proteins for pathological processes and inflammation. They are listed as follows: apoptotic marker cleaved caspase-3 (1:150, Cell Signaling Technology, Danvers, MA, USA); oxidative DNA damage marker 8-hydroxy-2'-deoxyguanosine (8-OHdG) (1:50, NOF Corporation, Tokyo, Japan); polymorphonuclear leukocytes (PMNL): MCA771GA (1:5000, AbD Serotec (Raleigh, NC, USA)); macrophages: F4/80 (1:100, Abcam, CA, USA); COX-2 (1:100, Cell Signaling Technology) and TNF-α (1:50, Santa Cruz, CA, USA). Negative controls included serum in lieu of primary antibody.

### Histomorphometry

The histomorphometric study was performed in a blinded manner on 30 slides (3 slides each placenta, 5 mice/placenta each group). PMNLs and macrophages were counted at 5 different random areas of placenta sections, which were taken from 5 mice (we lost one animal each from the original *P*.*g*.- infected and NC groups, n = 6 per group). The average number of cells per unit area was calculated for comparison.

### Cell Culture

A human trophoblast cell line (HTR-8/Svneo) was kindly provided by Dr. Charles H. Graham (Queen’s University, Canada). Cells were grown in RPMI 1640 media (Nissui Pharmaceutical Co., Tokyo, Japan) supplemented with 10% heat-inactivated FBS (Invitrogen) and 100 U/ml penicillin–streptomycin (Gibco, Tokyo, Japan). For experiments, cells were seeded at a density of 5 × 10^5^ cells in a 35 mm culture dish. Cells were maintained at 37°C in a normal atmosphere containing 5% CO_2_.

### Enzyme-linked immunosorbent assay (ELISA)

HTR-8 cells were treated with standard *P*.*g*.-LPS (1μg/ml), which was obtained from strain ATCC33277 (InvivoGen, SanDiego, CA, USA) and live and dead *P*.*g*. (w83 strain) at MOI 50 for 48 hours and protein levels of TNF-α in the supernatant were analyzed by Human TNF-α immunoassay (R&D Systems, Minneapolis, MN, USA) according to manufacturer’s instructions.

### RNA Isolation and RT-PCR Analysis

Total RNA was extracted from the mouse placental tissue using TRIzol reagent (Invitrogen, Tokyo, Japan) after manually grinding the tissue using sterile grinding stick. Total RNA was extracted from cell pellets (1.5 x 10^5^ cells) using the RNeasy Mini Kit (Qiagen, K.K., Tokyo, Japan) following the manufacturer’s instructions. GAPDH and 18S were used as internal reference genes for normalization. PCR primer sequences are listed in S1 Table.

### DNA Isolation and Nested PCR Analysis

Total DNA was extracted from gd 15 formalin-fixed, paraffin-embedded mouse placental tissue from *P*.*g*. and NC groups by TaKaRa DEXPAT (Shiga, Japan) according to the manufacturer’s instructions. To identify the *P*.*g*. W83 strain, we amplified the *mgl* gene expressed in the *P*.*g*. [17] encoding l-methionine-g deamino-eamercaptomethane-lyase (METase) by PCR. Genomic DNA of the *P*.*g*. W83 strain was used as a positive control. PCR products were used in nested PCR for 45 cycles (30 s denaturation at 94°C, 30 s annealing at 50°C, and 1 min extension at 72°C). Primer pairs used for nested PCR are listed in S1 Table (*P*.*g*.-mgl and *P*.*g*.-mgl-nested).

### Western Blotting

Western blotting was performed as described previously [18]. In brief, HTR-8 cell pellets (5 x 10^5^ cells) were resuspended in ice-cold lysis buffer. Proteins were separated by SDS-PAGE, electro-blotted onto nitrocellulose membrane, and were visualized by the ECL western blotting detection system (GE Healthcare, UK). Anti-phospho-JNK, anti-phospho-c-Jun, anti-JNK, anti-phospho-ERK1/2, anti-ERK1/2, anti-phospho-NF-κB p65 and anti-RelB antibodies (Cell Signaling Technology) and mouse monoclonal anti-β-actin (Sigma-Aldrich, St. Louis, MO, USA) were used as primary antibodies.

### Statistical Analysis

Statistical differences among experimental groups were evaluated by *t*-test or chi-square test using GraphPad Prism (version 6.0c, CA, USA) with the level of significance described for p < 0.01 (**) and p < 0.05 (*).

## Results

### Dental Infection of *P*.*g*. Increases Pro-inflammatory Cytokines in Serum (Fig 1)

**Fig 1:**
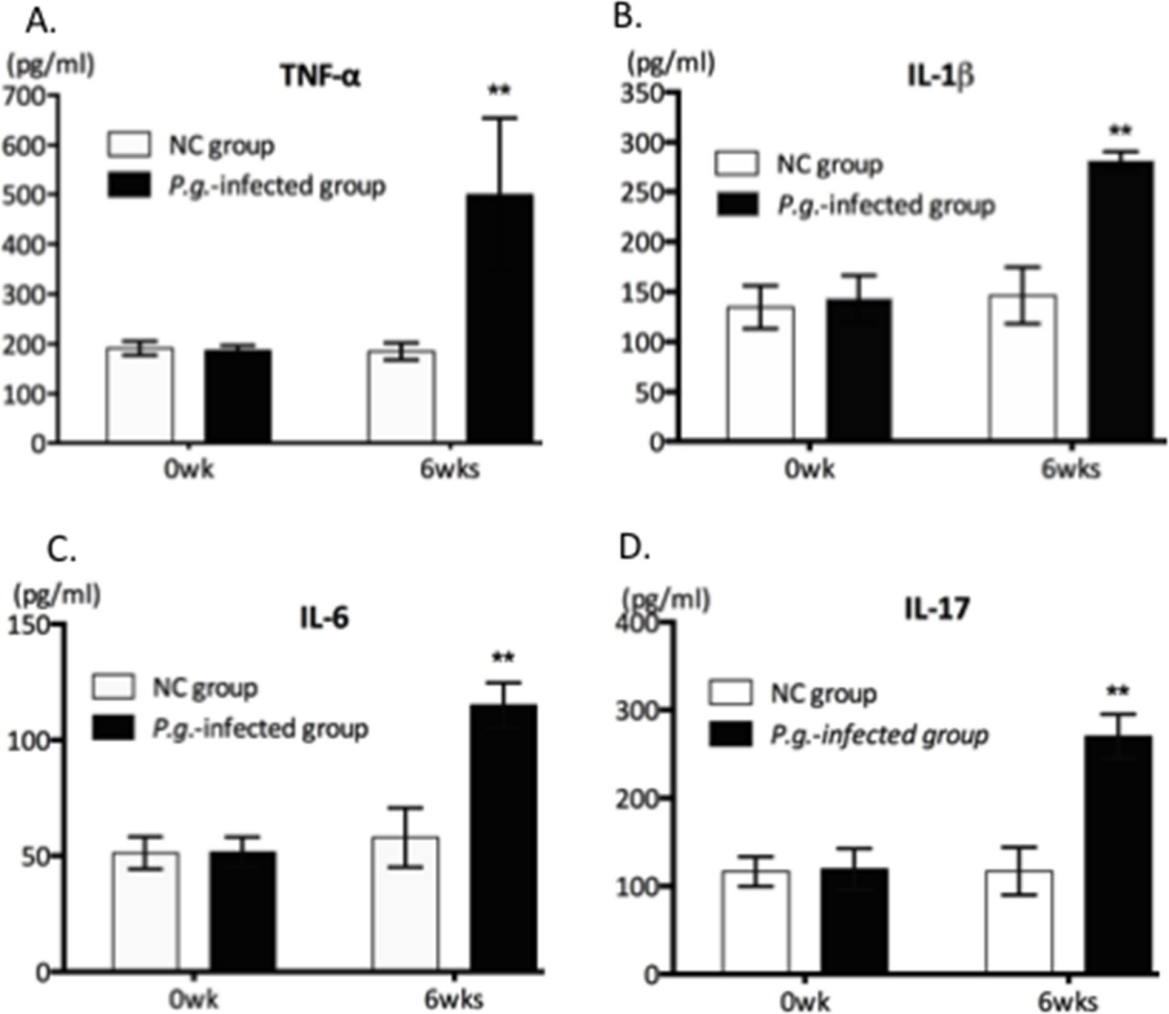
Dental Infection of *P*.*g*. Increases Pro-inflammatory Cytokines in Serum. ELISA analysis of TNF-α (A), IL-1β (B), IL-6 (C) and IL-17 (D) concentrations in serum (0wk or 6wks post-infection) of non-infected control (NC) and *P*.*g*.-infected groups (n = 6 for each). Statistical significance was determined using the Student t-test. **P<0.01.

We previously demonstrated that *P*.*g*. inoculation into the dental pulp induced local infection and periapical granuloma by 6 wks after infection [15]. Following *P*.*g*. dental infection, we investigated signs of systemic inflammation by measuring serum levels of pro-inflammatory cytokines at 6wks post-infection. ELISA analysis confirmed that the *P*.*g*.-infected group (n = 6) exhibited significantly increased circulating levels of pro-inflammatory cytokines including TNF-α (2.5-fold), IL-1β (2-fold), IL-6 (2-fold) and IL-17 (more than 2-fold) compared to NC (Fig 1), confirming that dental inoculation of *P*.*g*. induced systemic low-grade inflammation in this mouse model.

### Dental Infection of *P*.*g*. Induces PTB and LBW (Fig 2)

**Fig 2:**
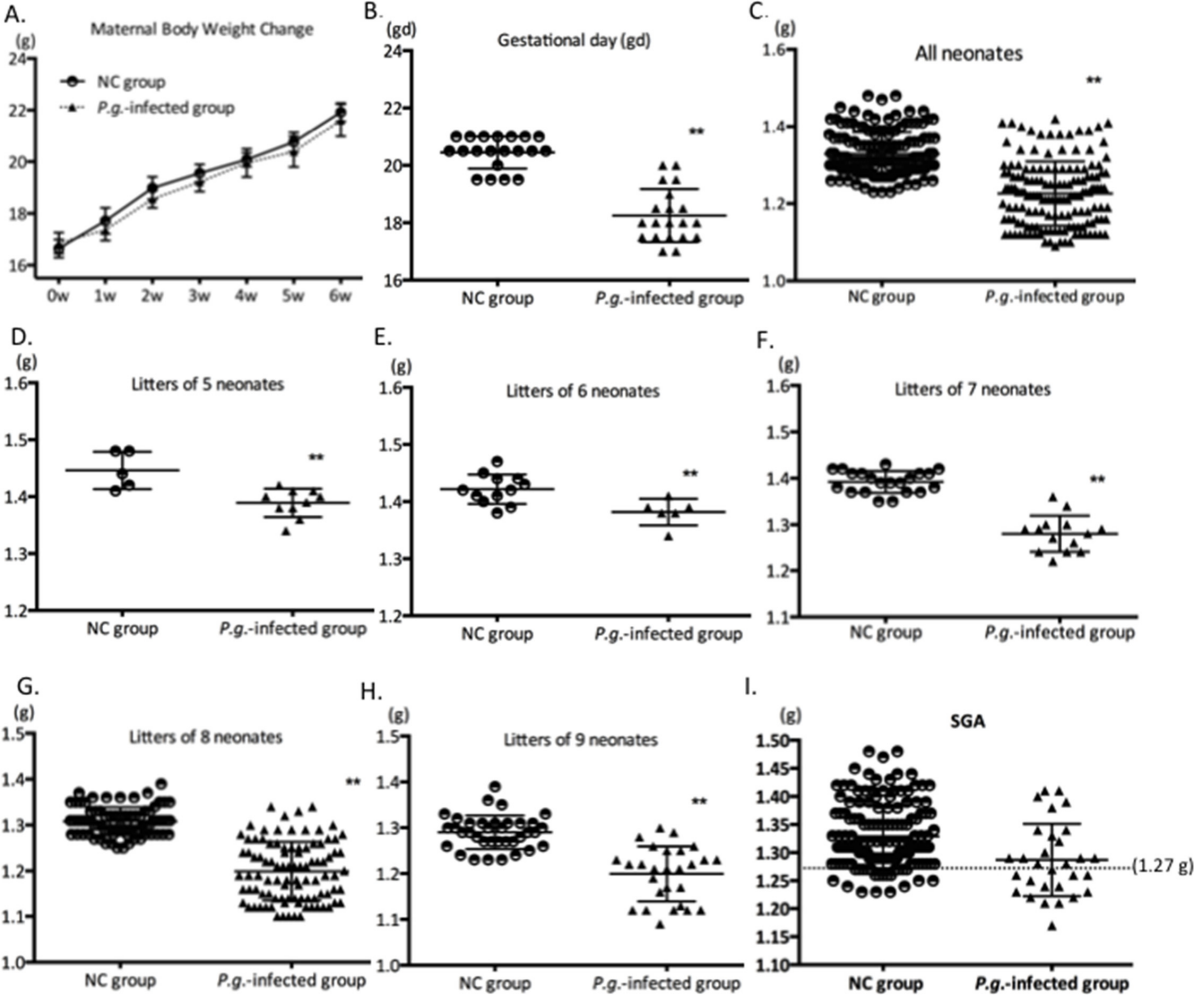
Dental Infection of *P*.*g*. Induces PTB and LBW. Maternal body weight changes (A) were recorded from *P*.*g*. infection (0 wks) to initiation of mating (6 wks post-infection). Gestational day (gd) (B) was confirmed in NC and *P*.*g*.-infected groups (n = 20 for each group). Means (±SD) are 20.45 ± 0.56 and 18.25 ± 0.925, respectively. Statistical significance was determined using the unpaired t-test. Birthweight of neonates (C-H) was compared between NC and *P*.*g*.-infected groups, considering all neonates or comparing litters with the same number of neonates (to control for litter size effect on birthweight). Statistical significance was determined using the unpaired t-test. SGA (small for gestational age) (I) was defined as pups birth weight less than the 10th percentile in NC group, which is 1.27 g (indicated by dotted line). 13 pups out of 30 pups in *P*.*g*.-infected groups were SGA. Statistical analysis for SGA is shown in Table 1. ** p < 0.01. *p < 0.05.

*P*.*g*. infection did not affect body weight over 6 weeks (Fig 2A). However, *P*.*g*. infection significantly altered (p < 0.01) the average gestational day (gd) of birth from gd 20.45 (in NC mice) to gd 18.25, considered to be PTB for mice (Fig 2B, S2 Table). Strikingly, some deliveries in the *P*.*g*. infected group were recorded at gd 17, close to extreme PTB for mice.

The average birth weight for all pups in the *P*.*g*.-infected group (1.23 g) was significantly lower than that of NC group (1.33 g) (p < 0.01) (Fig 2C). Additionally, the *P*.*g*.-infected group exhibited LBW regardless of the number of pups per litter (Fig 2C–2H). To verify the classification of LBW, either birth weight criteria or small-for-gestational age birth (SGA) criteria (birth weight below the 10th percentile for gestational age) was used [19]. According to human LBW criteria (S3 Table), the result of mice low birth weight (1.23 g) does not satisfy the calculated definition (< 0.97 g). In the *P*.*g*.-infected group, 30 pups (from 4 different litters) out of the 153 pups (19.6%) were born at term. They are 5 pups from 1 litter at gd 19.5; 9 pups from 1 litter at gd 19.5 and 8 pups from 2 litters at gd 20. Among those 30 pups, 13 pups (43%), are small-for-gestational age birth (SGA), which is 1.27 g determined by 10th percentile of NC group (Fig 2I, Table 1) (P<0.0001).

**Table 1:** Chi-Square analysis of SGA (small for gestational age) in *P*.*g*.-infected group *vs*. NC group.

Fisher’s exact test	<10th percentile	> = 10th percentile	Total
NC group	13	141	154
*P*.*g*.-infected group	13	17	30
Total	26	158	184

P<0.0001, two tailed

### Dental Infection of *P*.*g*. Induces Defects in Placental Tissues in Mice (Fig 3)

**Fig 3:**
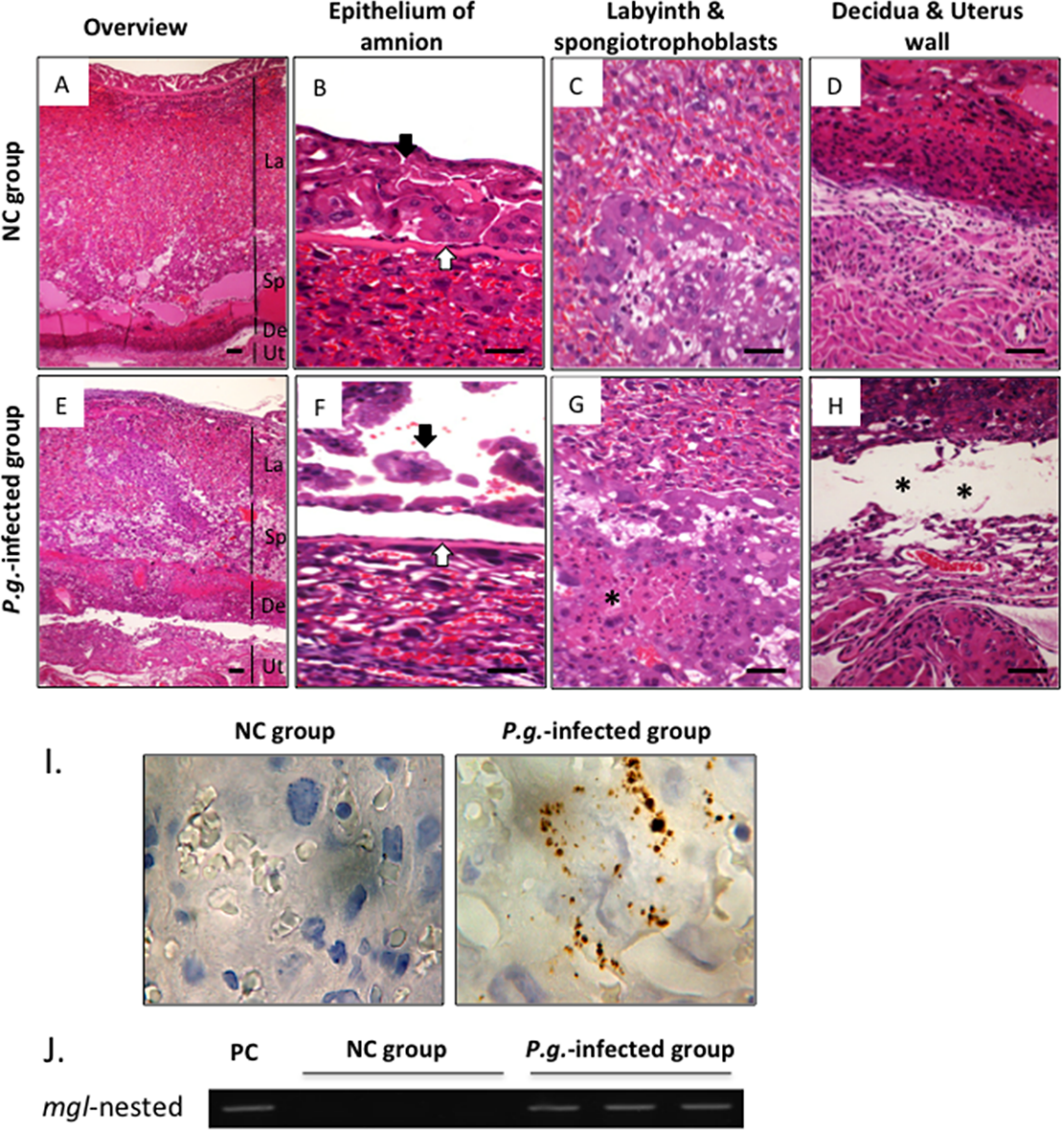
Dental Infection of *P*.*g*. Induces Defects in Placental Tissues in Mice. Representative histological findings in gd 15-placental tissue from the NC group (A-D) and *P*.*g*.-infected group (E-H) by H&E staining (n = 6 for each group). (A, E) Overview of mouse placenta: La, Labyrinth; Sp, Spongiotrophoblasts; De, Decidua; Ut, Uterus wall. (B, F) Epithelium of amnion: in the *P*.*g*.-infected group, amnion epithelium (black arrow) is degenerative and detached from chorionic plate (white arrow). (C, G) Labyrins and Spongiotrophoblasts layers: Trophoblasts and endothelial cells are necrotic (*) in *P*.*g*.-infected group. (D, H) Decidua and uterus wall: Placental abruption is evident in the *P*.*g*.-infected group. ** shows the separation between placenta and uterus at maternal-fetus junction. (I) Immunolocalization of *P*.*g*. (brown pigments) (n = 6 for each group); 1000x-magnification by oil-immersed microscopy. (J) Gene expression for the *mgl*-gene of *P*.*g-*W83 strain by nested PCR; representative results from 6 mice for each group. PC, positive control. Scale bars, 100μm. Olympus BH2 microscope and Nikon digital sight DS-L2 camera were used for capturing images.

We analyzed the histology of placental tissues obtained on gd 15 in order to identify local changes caused by *P*.*g*. infection that may contribute to PTB/LBW. In normal placental tissue in the NC group, the amnion was still intact and was covering the chorionic plate (Fig 3A and 3B); Intact spongiotrophoblasts, labyrinth trophoblasts and endothelial cells were observed (Fig 3C) and the decidua (uterine lining, or endometrium) was firmly attached to the uterus (Fig 3D). In contrast, in placental tissues in the *P*.*g*. infected group, the amnion was degenerative, with partial detachment from the surface of chorionic plate (Fig 3F), meeting the criteria for premature rupture of membrane (PROM), a contributing factor to PTB. Furthermore, in the *P*.*g*. infected group, trophoblasts and endothelial cells appeared degenerative and necrotic areas were observed (Fig 3G), and the placenta was detached from the uterus (Fig 3H), a condition known as placental abruption that can affect both the mother and fetus.

In order to better understand these changes in placental tissues, we next examined whether *P*.*g*. was detectable in placental tissues. By immunohistochemistry, no *P*.*g*. was detected in the NC group, however, colonies were detected in placental tissues of the *P*.*g*-infected group (Fig 3I). Nested PCR confirmed the presence of the *mdl*-gene encoding a *P*.*g*.-specific enzyme (Fig 3J).

### Increased Inflammatory Cell Infiltration and Inflammatory Mediators in Placental Tissues Following *P*.*g*. Infection (Fig 4)

**Fig 4:**
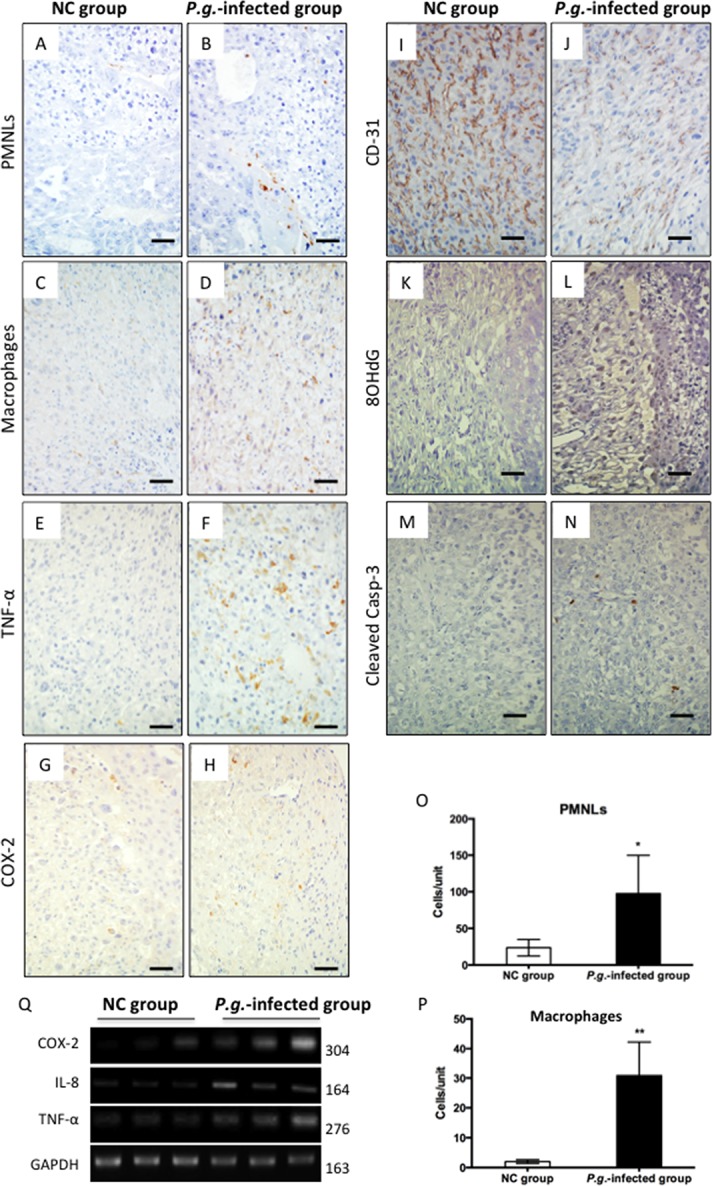
Increased Inflammatory Cell Infiltration and Inflammatory Mediators in Placental Tissues Following *P*.*g*. Infection. Immunohistochemical staining of gd 15 placental tissues of NC and *P*.*g*.-infected groups (n = 6 for each group). (A,B) PMNLs, (C,D) F4/80 positive macrophages, (E,F) TNF-α positive macrophages, (G, H) COX-2 positive staining, (I, J) CD-31 stained endothelial cells, (K,L) 8OHdG (oxidative DNA stress marker), (M,N) cleaved-caspase 3 (apoptosis marker), and (O,P) histomorphometric analysis of PMNLs and F4/80 (macrophages). Scale bars, 100μm. Statistical significance was determined using the Student t-test. *p < 0.05, **p < 0.01. Olympus BH2 microscope and Nikon digital sight DS-L2 camera were used for capturing images. (Q) Representative results of mRNA expression of COX-2, Gal-3, IL-8 and TNF-α in placental tissues in NC group and *P*.*g*.-infected group (n = 3 for each), GAPDH was used as internal control. Molecular weights were labeled to the right of each band. Yellow dashed square shows that PMNLs are restricted to the blood vessels.

Labor at term involves physiological inflammatory processes including infiltration of leukocytes and production of proinflammatory cytokines (Oaman et al. 2003). In order to identify changes in these processes related to *P*.*g*. infection, we used immunohistochemistry to analyze the presence and location of inflammatory cells including polymorphonuclear leukocytes (PMNLs) and macrophages. In the NC group, PMNLs were restricted in the blood vessel wall in decidua (Fig 4A), whereas in the *P*.*g*.-infected group, PMNLs were recruited to the area of trophoblasts (Fig 4B), suggesting increased infiltration of cells. The numbers of both PMNLs (p < 0.05, Fig 4O) and macrophages (p < 0.01, Fig 4P) were significantly increased in placental tissues of the *P*.*g*.-infected group in comparison with the NC group. While pro-inflammatory mediators including TNF-α and COX-2 were barely detectable in NC placental tissues (Fig 4E and 4G), numerous TNF-α (Fig 4F) and COX-2 (Fig 4H) positive cells were detected in tissues of the *P*.*g*.-infected group.

Due to the prominent degenerative changes observed in placental tissues in the *P*.*g*. infected group (Fig 3), we examined markers of cellular damage in endothelial cells and trophoblasts. Immunostaining for CD-31, a marker of endothelial cells, revealed consistently strong expression in endothelial cells in the NC group (Fig 4I). However, much weaker and discontinuous staining was observed in the *P*.*g*.-infected group (Fig 4J), indicating endothelial cell damage. Further pathological changes in the *P*.*g*.-infected group were suggested by increased Immunostaining of both 8OHdG (an indicator of oxidative DNA damage) and cleaved caspase-3 (one of the key executioners of apoptosis)(Fig 4K–4N). PCR analysis of placental tissues confirmed detectable up-regulation of COX-2, IL-8 and TNF-α mRNA in the *P*.*g*.-infected group (Fig 4Q)

### *P*.*g*. LPS Up-regulates Expression of Inflammatory Mediators via NF-κB Signaling in Trophoblasts (Fig 5)

**Fig 5:**
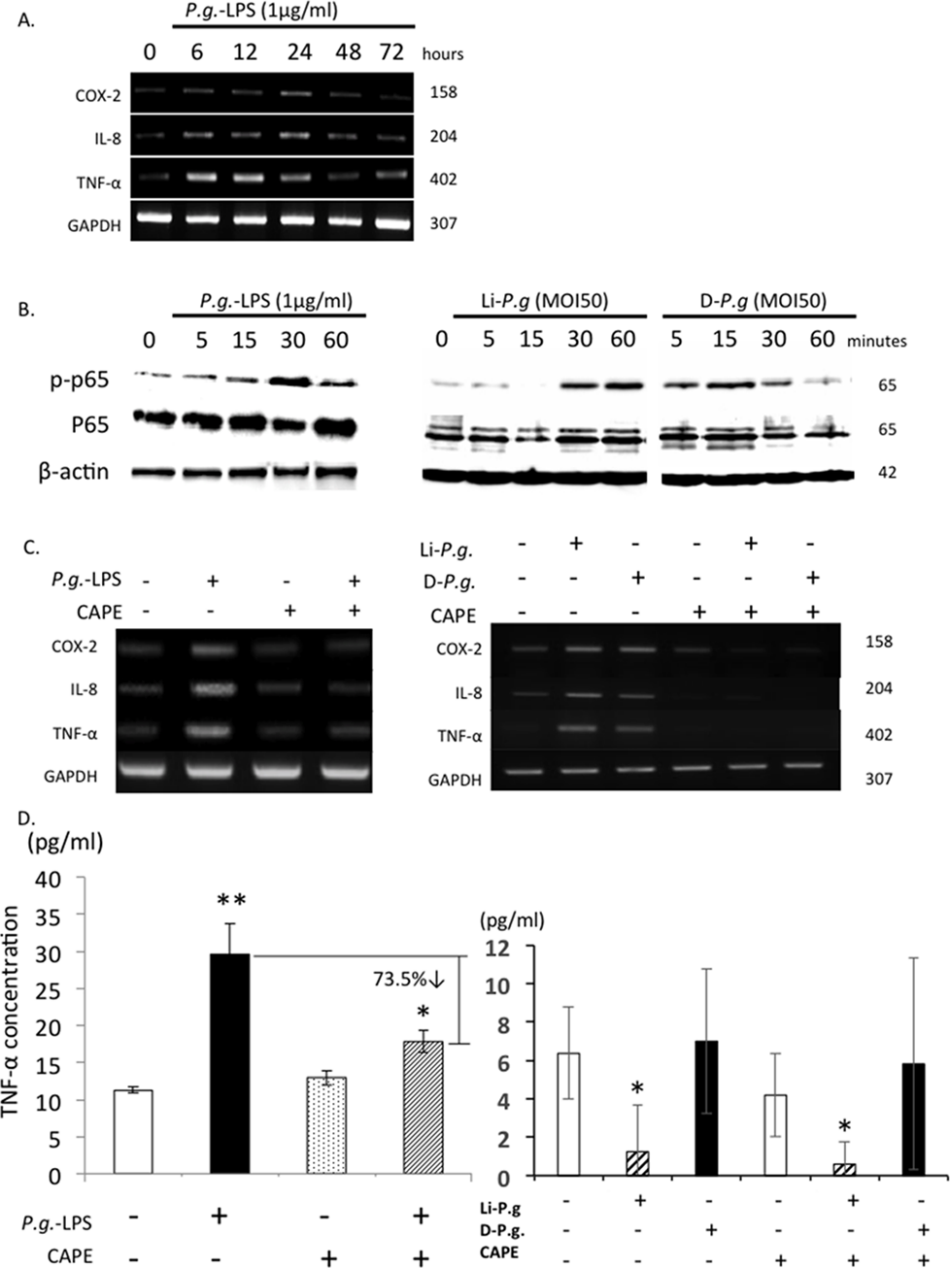
*P*.*g*. LPS Up-regulates Expression of Inflammatory Mediators via NF-κB Signaling in Trophoblasts. (A) HTR-8 trophoblasts were seeded (5x10^5^ cells/well) in 6-well culture plates and culture media were changed once before stimulation. Cells were treated by *P*.*g*.-LPS (1 μg/ml unless otherwise noted) and both culture medium and cells were collected. mRNA expressions of COX-2, IL-8 and TNF-α were analyzed from cell pellets. (B) HTR-8 cells were stimulated with *P*.*g*.-LPS, Li-*P*.*g*. (live-*P*.*g*.) or D-*P*.*g*. (dead-*P*.*g*.) and cell lysates were examined by immunoblotting analysis using p-p65, p65. Molecular weight is labeled to the right of each band. (C,D) HTR-8 cells were pretreated with or without CAPE for 4 hrs and then stimulated with or without *P*.*g*.-LPS, Li-*P*.*g*. (live-*P*.*g*.) or D-*P*.*g*. (dead-*P*.*g*.) for 24 hrs. mRNA expressions of COX-2, IL-8 and TNF-α (C); protein secretion of TNF-α were examined (D). TNF-α amount in negative controls were subtracted as basal levels when calculating the percentage of down-regulation. GAPDH or β-actin was used as internal control. MOI, multiplicity of infection. *p < 0.05, **p < 0.01. Experiments were performed at least three times with similar results.

The pathological changes observed in the *P*.*g*. infected group strongly suggest the effects of bacteria and their products on local inflammation in placental tissues. We analyzed direct effects of *P*.*g*. lipopolysaccharide (LPS), on HTR-8 trophoblasts, *in vitro*. Addition of 1 μg/ml *P*.*g*.-LPS transiently upregulated mRNA expression of inflammatory mediators COX-2, IL-8 and TNF-α, with peak expression of COX-2 and IL-8 at 24 hrs, and peak expression of TNF-α at 6 hrs (Fig 5A).

Addition of *P*.*g*.-LPS also increased phosphorylation of p65, a transcription factor associated with NF-κB activation, with maximum intensity at 30 min (Fig 5B). Pretreatment of HTR-8 cells with CAPE, an inhibitor of NF-κB activation, inhibited LPS-induced up-regulation of COX-2, IL-8 and TNF-α (Fig 5C), and significantly reduced TNF-α protein secretion by 73.5% (Fig 5D), confirming that NF-κB activation contributes to these changes in inflammatory mediators. On the other hand, live *P*.*g*. infection and dead *P*.*g*. treatment also induced p-p65 activation peaking at 30–60 min and 5 min respectively (Fig 5B). Inhibition of NF-κB activation with CAPE also down-regulated live-*P*.*g*. and dead-*P*.*g*. induced COX-2, IL-8 and TNF-α mRNA expression (Fig 5C). However up-regulated production of TNF-α protein was not evident. Interestingly, live P.g. infection induced significant reduction of TNF-α with/without CAPE treatment (Fig 5D).

## Discussion

Epidemiological studies have revealed a link between dental infection and preterm birth or low birth weight (PTB/LBW). In order to identify underlying mechanisms linking periodontitis and PTB/LBW, we established a mouse model of chronic periodontitis during pregnancy. We demonstrated that dental pulp infection in mice with keystone periodontal pathogen *P*. *gingivalis* (*P*.*g*.) significantly increased circulating pro-inflammatory cytokines, including TNF-α, IL-1β, IL-6 and IL-17. We found that *P*.*g*. infection predisposed to significantly earlier birth and lower birth weights. *P*.*g*. was localized to placental tissues by immunohistochemistry and PCR, and defects in placental tissues of *P*.*g*.- infected mice were documented, including premature rupture of membrane (amnion), placental detachment, degenerative changes in trophoblasts and endothelial cells, and necrotic areas. *P*.*g*. infection significantly increased expression of polymorphonuclear leukocytes (PMNLs) and macrophages in placental tissues, associated with increased local expression of pro-inflammatory mediators including TNF-α and COX-2. Further placental tissue damage was indicated in *P*.*g*. infected mice by decreased CD-31 in endothelial cells, increased expression of 8OHdG, an indicator of oxidative DNA damage, and cleaved caspase-3, a marker of apoptosis. Lastly we showed that *P*.*g*. lipopolysaccharide (LPS) induced significantly increased expression of COX-2, IL-8 and TNF-α, in HTR-8 trophoblasts in an NF-κB-dependent fashion, supporting potential for *P*.*g*. to have direct pro-inflammatory effects on local cells in the placenta.

### Dental infection as a source for systemic inflammation

In the present study, we used a mouse model where dental infection was established by direct inoculation with *P*.*g*., and then mice were mated after 6 wks to determine effects of periodontal infection on gestational day, birth weight, and placental tissues. *P*.*g*. is keystone pathogen associated with chronic periodontitis, requiring anaerobic conditions for growth, and is detected in infected pulp with periapical periodontitis [20,21]. In order to mimic “natural” routes of local infection by *P*.*g*., we directly applied bacterial cultures into molar pulp chambers and allowed 6 weeks for bacterial colonies to become established and potentially spread. Using this model, we previously confirmed that *P*.*g*. remained viable within the pulp chamber, proliferated over an extended period, induced periapical periodontitis, and elevated circulating LPS levels [15]. In this study, we documented significantly increased pro-inflammatory cytokines including TNF-α, IL-17, IL-6 and IL-1β in serum at 6 wks post-infection, at which time mating occurred. These combined data all support the assertion that dental infection with *P*.*g*. was capable of inducing long-term, low-grade systemic inflammation in mice, which parallels the clinical condition of pregnant women with periodontitis [22].

Previous studies have used different approaches to assess the effects of dental pathogens on pregnancy outcomes. For example, Hansen et al. reported that intravenous injection of *F*. *nucleatum* (10^5^ or 10^6^ CFU) to mice at gd16/17 induced fetal death by 48 to 72 hrs post-injection [23]. Exposure to *P*.*g*. from the reproductive tract in hamsters was also capable of inducing PTB or LBW [24]. Additionally, *P*.*g*. (10^7^ CFU, A7436 strain) infection in mice induced fetal growth restriction at gd16.5 [25]. All of the above animal models had the limitations of being short-term approaches for examining the relationship between infection of oral bacteria and pregnancy outcome, and not mimicking the natural route of infection initiating in the dental tissues. To our knowledge, this study is the first to substantiate the association between a chronic focal infection, in this case dental infection with periodontal pathogen *P*.*g*., and adverse pregnancy outcomes of PTB/LBW.

### Effects of dental infection on gestational day and birth weight

Using the animal model described in these studies, we confirmed that *P*.*g*. infected mice exhibited PTB (by an average of 2 days) and comparative LBW. Although the LBW in mice (1.23 g) recorded here does not satisfy the calculated definition according to human LBW (0.97 g), a statistically significant difference (p < 0.01) was observed compared with controls. Nath et al. reported that significantly more mice exhibited PTB when injected following intrauterine injections with *E*.*coli* (10^4^ CFU) at gd 14.5, where PTB was defined as prior to gd 18.5 [26]. In the present study, 81.4% of *P*.*g*.-infected mice delivered prior to gd 18.5, comparable to results from direct intrauterine injection of *E*.*coli* (10^4^ CFU). Surprisingly, dental infection of *P*.*g*. induced not only LBW associated with PTB, but also small-for-gestational-age (SGA), indicating more severe fetal growth restriction. Among those pups born at term in the *P*.*g*.-infected group, 43% of their birth weights were below the 10th percentile compared to the NC group. The 10^th^ percentile is a typical criterion for the determination of SGA classification, and is associated with increased risk of perinatal morbidity and mortality [19,27,28].

LBW in infants (including SGA) directly or indirectly contributes to 60–80% of neonatal deaths and increased risk of early growth retardation, infectious disease, developmental delay, and death during infancy and childhood. Because of this documented morbidity and mortality associated with PTB/LBW, treatment of dental infections, including marginal and periapical periodontitis (most common infectious disease of mankind), would be expected to have a beneficial impact on reducing PTB/LBW and improving of maternal and neonatal quality of life.

### Mechanisms for effects of dental infection on pregnancy outcomes

The underlying biological mechanisms for induction of PTB/LBW as a result of dental infection are proposed to include 1) translocation of periodontal pathogens, 2) increased levels of LPS, 3) production of inflammatory mediators and 4) local changes in placental tissues. Using this mouse model of dental *P*.*g*. infection followed by pregnancy, combined with *in vitro* experiments, we demonstrate all four of these events.

*P*.*g*. introduced into the dental pulp was later detected (during pregnancy) in mouse placental tissues by immunohistochemistry and PCR. Similarly, our previous study demonstrated immunolocalization of *P*.*g*. colonies in hepatocytes and Kupffer cells in the liver tissues of *P*.*g*. odontogenic infected mouse model [15]. In addition, subcutaneous infection of *P*.*g*. led to detectable colonies in the maternal liver or placenta in mice or rabbits [25,29]

It is well accepted that LPS-induced local inflammation induces PTB through TLR4 signaling [30]. LPS is a critical virulence factor of *P*.*g*. [31,32], and we previously reported elevated circulating LPS levels in *P*.*g*. infected mice [16]. Therefore, we further examined the effect of *P*.*g*.*-*LPS on HTR-8 trophoblasts in comparison with cells stimulated by *E*.*Coli*-LPS (conventional enteric bacterial LPS) or *A*.*a*.*-*LPS (another periodontal pathogen LPS) in vitro. We demonstrated that *P*.*g*.-LPS stimulation significantly increased expression of pro-inflammatory mediators COX-2, IL-8 and TNF-α through NF-κB pathway as did *E*.*Coli*-LPS and *A*.*a*.*-*LPS (S1 Fig). COX-2 is an inducible enzyme responsible for the synthesis of inflammation-related prostaglandins (PGs). PGs play a central role in activation of myometrium to induce PTB [33]. TNF-α is a well-known inflammatory mediator and is suggested to be a predictor of premature rupture of membrane (PROM) and cervical ripening [34]. IL-8 is associated with recruitment of neutrophils and induces a further increase in oxidant stress mediators related with PTB [35]. These collected data support that *P*.*g*.-LPS induced local inflammation induced via the TLR2/TLR4-NF-κB pathway has a critical role in PTB caused by odontogenic infection of *P*.*g*. Moreover, we also confirmed that *A*.*a*.-LPS induced proinflammatory cytokine production from trophoblasts (S1 Fig). It has been reported that *A*.*a*., another periodontal pathogens, induced trophoblasts apoptosis in vitro [36]. Additional studies are needed to clarify the downstream effects from dental infections involving other periodontal pathogens.

We also examined the effects of live *P*.*g*. infection and dead *P*.*g*. application, which may reach to placenta through circulation and challenge to the trophoblasts. Interestingly,

Both live and dead *P*.*g*. up-regulated TNF-α, IL-8 and COX-2 at the mRNA level though TNF-α production was not detected at the protein level. In addition, live *P*.*g*. significantly reduced TNF-α production. While speculative, it is possible that live *P*.*g*. expressed proteases like gingipains may contribute to reduction of TNF-α through degradation of cytokines [37]. In the present study, we confirmed that *P*.*g*.*-*LPS in circulation might be an important virulence factor for PTB caused by odontogenic infection. Further studies are required to determine the effects of *P*.*g*. infection on trophoblasts.

Critically, we demonstrate multiple consequences of *P*.*g*. infection in placental tissues, including detachment of the amnion from the surface of chorionic plate degenerative changes in trophoblasts and endothelial cells, detachment of the placenta from the uterus, increased infiltration of inflammatory cells including PMNLs and macrophages, increased localization of TNF-α, COX-2, and IL-8, and evidence of pathological changes including oxidative DNA damage and apoptosis.

## Conclusions

Our novel mouse model described here supports epidemiological data in humans that dental infection predisposes to PTB/LBW. We demonstrate PTB and LBW in infected mice, translocation of *P*.*g* to placental tissues, increased circulating and local pro-inflammatory markers, and the capability of *P*.*g*. LPS to directly induce cytokine production in trophoblasts, *in vitro*. These findings further underscore the importance of local and systemic infections and inflammation during pregnancy and suggest that prevention and/or elimination of dental infections such as periodontitis before pregnancy may have a beneficial effect on PTB/LBW.

## Supporting Information

S1 Fig HTR-8 trophoblasts were seeded (5x10^5^ cells/well) in 6-well culture plates and culture media were changed once before stimulation. Cells were treated by *E*.*Coli*-LPS (0.1 μg/ml) or *A*.*a*.-LPS (0.1 μg/ml) and both culture media and cells were collected. mRNA expression of COX-2, IL-8 and TNF-α was analyzed from cell pellets. *E*.*coli*, *Escherichia coli; A*.*a*., *Aggregatibacter actinomycetemcomitans*. GAPDH was used as internal control. Experiments were performed at least three times with similar results.
(TIF)

S1 Table Sequences of primer pairs. Genes tested are listed according to human, mouse and microbial categories. Sequences of primer pairs, product size and gene accession numbers are indicated respectively.
(TIF)

S2 Table Definition of preterm birth in humans and mice. Full term pregnancy in humans last an average of 40 weeks. Preterm birth is defined as live birth before 37 weeks of gestation and extreme preterm birth is defined as live birth before 32 weeks of gestation [19]. In our study, full term pregnancy in control mice lasted an average of 20.5 days. By correlating defined pregnancy times points in humans, we determined that preterm birth in mice is before 18.5 days and extreme preterm birth is before 16.5 days.
(TIF)

S3 Table Definition of low birth weight humans and mice. Average birth weight in humans is 3400 g and low birth weight is less than 2500 g [19]. In our study, average birth weight of pups in control group was 1.33 g. By correlating defined birth weight in humans, we determined that low birth weight in mice is less than 0.97 g.
(TIF)
