# Supplementary figures and images for: Dental Infection of Porphyromonas gingivalis Induces Preterm Birth in Mice
Source: PLoS One. 2015 Aug 31;10(8):e0137249. doi: 10.1371/journal.pone.0137249 (PMC4556457; doi:10.1371/journal.pone.0137249)

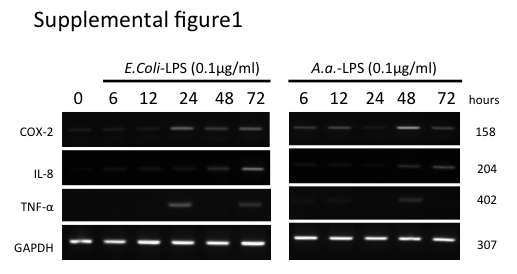

Supplement: S1 Fig — Cells were treated by E.Coli-LPS (0.1 μg/ml) or A.a.-LPS (0.1 μg/ml) and both culture media and cells were collected. mRNA expression of COX-2, IL-8 and TNF-α was analyzed from cell pellets. E.coli, Escherichia coli; A.a., Aggregatibacter actinomycetemcomitans. GAPDH was used as internal control. Experiments were performed at least three times with similar results. (TIF) [file pone.0137249.s001.tif]

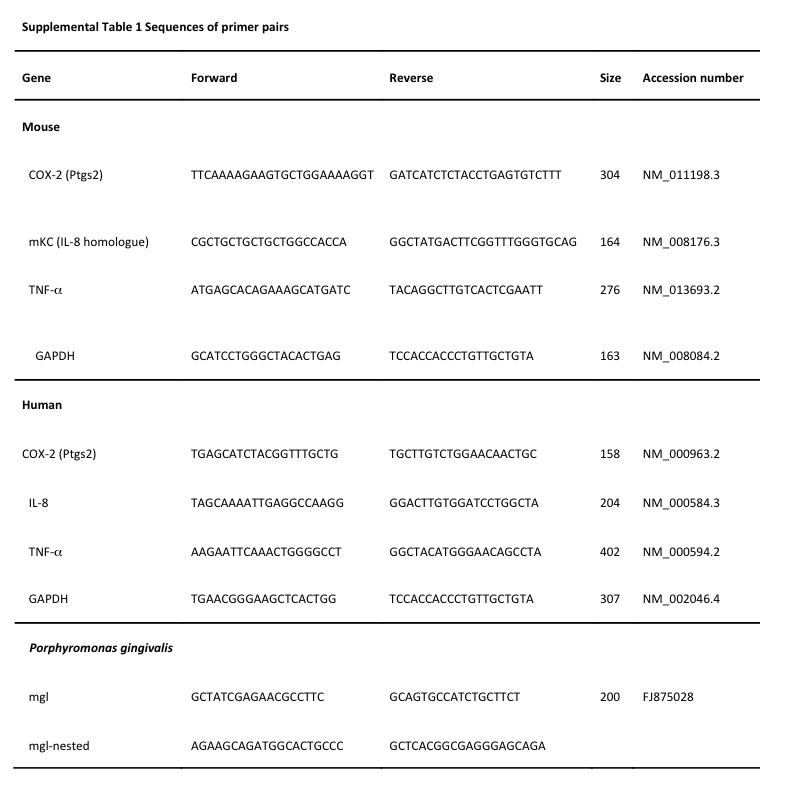

Supplement: S1 Table — Genes tested are listed according to human, mouse and microbial categories. Sequences of primer pairs, product size and gene accession numbers are indicated respectively. (TIF) [file pone.0137249.s002.tif]

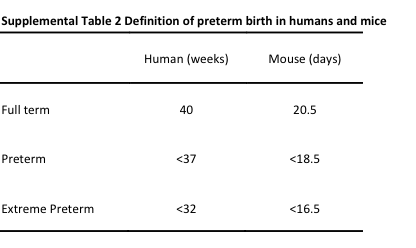

Supplement: S2 Table — Full term pregnancy in humans last an average of 40 weeks. Preterm birth is defined as live birth before 37 weeks of gestation and extreme preterm birth is defined as live birth before 32 weeks of gestation [19]. In our study, full term pregnancy in control mice lasted an average of 20.5 days. By correlating defined pregnancy times points in humans, we determined that preterm birth in mice is before 18.5 days and extreme preterm birth is before 16.5 days. (TIF) [file pone.0137249.s003.tif]

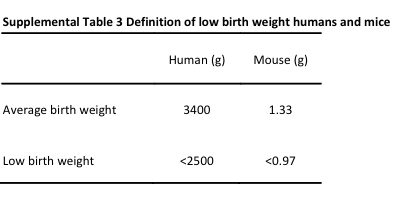

Supplement: S3 Table — Average birth weight in humans is 3400 g and low birth weight is less than 2500 g [19]. In our study, average birth weight of pups in control group was 1.33 g. By correlating defined birth weight in humans, we determined that low birth weight in mice is less than 0.97 g. (TIF) [file pone.0137249.s004.tif]
